# GABAergic signaling by cells of the immune system: more the rule than the exception

**DOI:** 10.1007/s00018-021-03881-z

**Published:** 2021-06-21

**Authors:** Amol K. Bhandage, Antonio Barragan

**Affiliations:** Department of Molecular Biosciences, The Wenner-Gren Institute, Stockholm University, Stockholm, Sweden

**Keywords:** Neurotransmission, Inflammation, Macrophage, *Toxoplasma*, Apicomplexa, Host–pathogen, Voltage-dependent calcium channel, Cation-chloride cotransporter

## Abstract

Gamma-aminobutyric acid (GABA) is best known as an essential neurotransmitter in the evolved central nervous system (CNS) of vertebrates. However, GABA antedates the development of the CNS as a bioactive molecule in metabolism and stress-coupled responses of prokaryotes, invertebrates and plants. Here, we focus on the emerging findings of GABA signaling in the mammalian immune system. Recent reports show that mononuclear phagocytes and lymphocytes, for instance dendritic cells, microglia, T cells and NK cells, express a GABAergic signaling machinery. Mounting evidence shows that GABA receptor signaling impacts central immune functions, such as cell migration, cytokine secretion, immune cell activation and cytotoxic responses. Furthermore, the GABAergic signaling machinery of leukocytes is implicated in responses to microbial infection and is co-opted by protozoan parasites for colonization of the host. Peripheral GABA signaling is also implicated in inflammatory conditions and diseases, such as type 1 diabetes, rheumatoid arthritis and cancer cell metastasis. Adding to its role in neurotransmission, growing evidence shows that the non-proteinogenic amino acid GABA acts as an intercellular signaling molecule in the immune system and, as an interspecies signaling molecule in host–microbe interactions. Altogether, the data raise the assumption of conserved GABA signaling in a broad range of mammalian cells and diversification of function in the immune system.

## Introduction

Gamma-aminobutyric acid (GABA) was first identified in 1949 as a plant metabolite [1] and, shortly after (1950), it was reported in the vertebrate brain [2]. Today, GABA has an undisputed role as the principal inhibitory neurotransmitter in the central nervous system (CNS) of vertebrates [3]. Yet, GABA has also metabolic and signaling functions in prokaryotic and eukaryotic microorganisms, and in invertebrates [4]. It is also well established that GABA has functions in the peripheral nervous system [5, 6]. More recently, GABA has been found in pancreatic islets [7] and peripheral GABAergic signaling has been implicated in cancer and other inflammatory conditions in humans [8–11].

Neurons and other GABAergic cells synthesize GABA via glutamate decarboxylases (GAD65/67) and transamination (GABA-T) catabolizes GABA [12]. GABA is released from cells by exocytosis or shuttled in and out of cells via GABA transporters (GATs) [13]. Upon extracellular release, GABA can activate GABA-A receptors (GABA-A Rs) [14] and GABA-B Rs [15] located in the cell membrane. GABA-B receptors are metabotropic G-protein-coupled receptors, while GABA-A Rs are pentameric ionotropic chloride channels, normally comprised of three types of subunits: 2 *α*’s, 2 *β*’s, and a third type of subunit. By combining the 19 different mammalian GABA-A R subunits (*α*1–6, *β*1–3, *γ*1–3, *δ*, *ε*, *π*, *θ* and *ρ*1–3), numerous variants of heteropentameric receptors can form in neuronal cells. Additionally, the *ρ* subunits can form homopentameric channels [16].

The strength and polarity of GABA signaling by GABA-A Rs is modulated by cation-chloride cotransporters (CCCs), that regulate intracellular chloride (Cl^−^) concentrations among other functions [17]. CCCs maintain the Cl^−^ gradient to favor outward Cl^−^ flux (Na–K–Cl cotransporters, NKCCs) or inward Cl^−^ flux (K–Cl cotransporters, KCCs). GABA-A R activation by GABA can elicit opening of voltage-dependent calcium (Ca^2+^) channels (VDCCs) with subsequent calcium Ca^2+^ influx into the neuronal cell [18]. GABA-A Rs have a broad range of sensitivity. Synaptic receptors are activated by millimolar concentrations of GABA, whereas extra-synaptic or non-synaptic receptors can be activated by GABA concentrations in the picomolar range [19–21]. In peripheral tissues and blood, sub-micromolar GABA concentrations have been measured [22–24]*.*

In vertebrates, immune cells derive from common progenitor stem cells in the bone marrow, which generate myeloid lineages [monocytes, dendritic cells (DCs), macrophages, granulocytes], or lymphoid lineages [B, T, natural killer (NK) cells] [25]. These cells mediate the complex responses that entail combating infections, cancer and tissue injury. The first line of defense is the innate response which is immediate. The second line of defense is the adaptive immune response which is generally highly specific and long-lasting. Naturally, the orchestration of innate and adaptive immune responses requires a tight regulation within the immune system and entail ever-broadening signaling cascades [26]. Mounting evidences show that immune cells can respond to neurotransmitters, for example acetylcholine [27], and signaling molecules present in the CNS are emerging as modulators of immune function [28]. Here, we outline recent findings on the role of GABA signaling in immune cells and discuss its impact on the effector functions of immune cells and disease.

## GABAergic signaling in mononuclear phagocytes

The mononuclear phagocyte system comprises DCs, monocytes, macrophages and brain microglia, among others [29]. Mononuclear phagocytes have diverse immunological functions and are crucial to counteract microbial infection. As sensors and effector cells in peripheral tissues, phagocytes participate in phagocytosis, cytokine responses and antigenic presentation for initiation of adaptive immune responses. The trafficking of phagocytes in response to external cues, for example invasive pathogens, is complex and the molecular signaling that regulates migration has not been fully elucidated [30]. Chemokine signaling cues guide afferent responses to inflammation sites and efferent responses, for example migration of DCs to lymph nodes where adaptive immune responses are initiated [31]. To avoid clearance by the immune response, pathogens have evolved diverse strategies to subvert this fundamental function of DCs and other mononuclear phagocytes [32, 33]. Paradoxically, these fundamental host-protective immune responses also constitute a gate for immune evasion and dissemination by pathogens.

### GABAergic signaling components expressed by mononuclear phagocytes

**Fig. 1:**
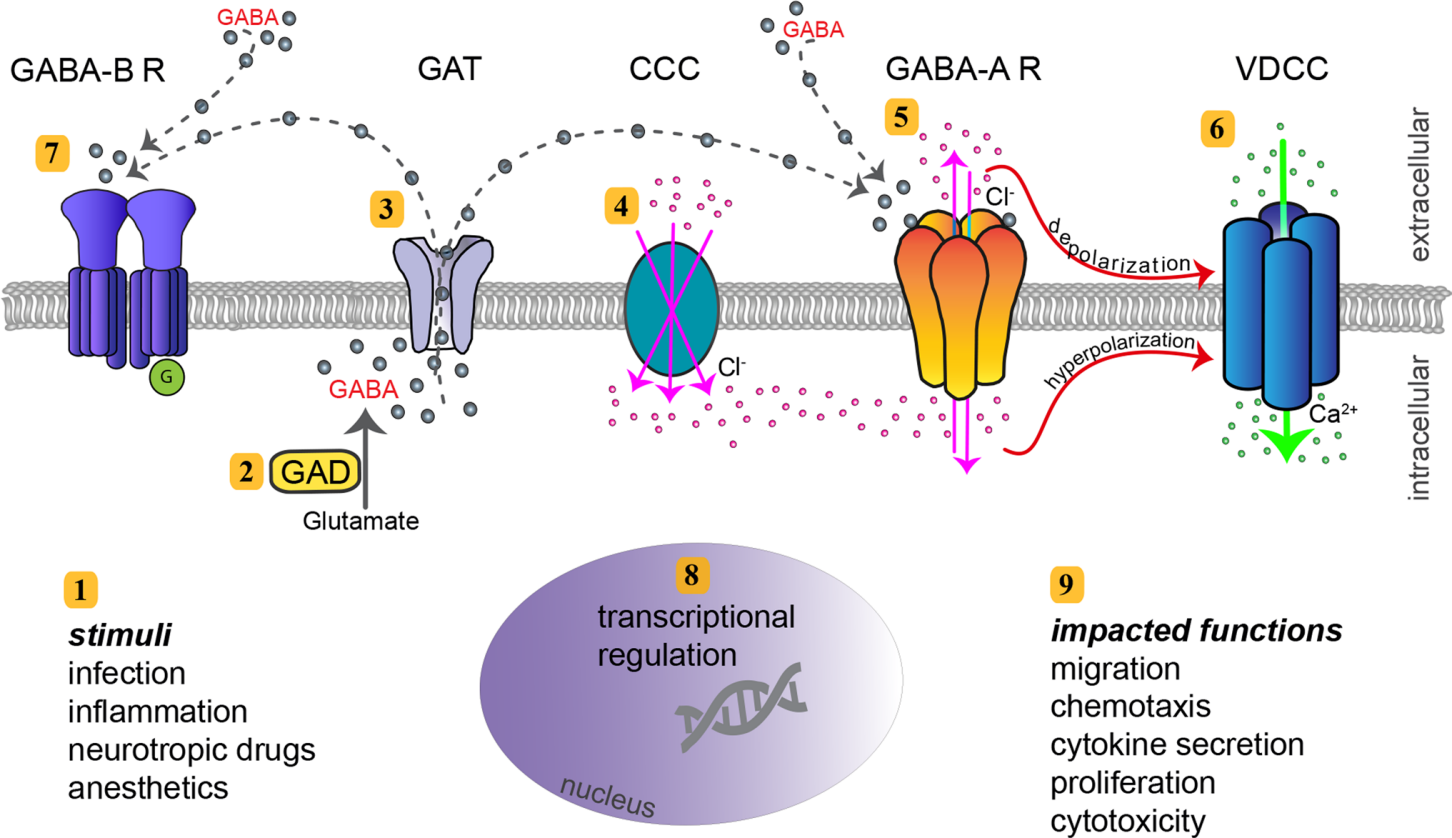
Molecular GABA signaling components, and immune cell functions linked to GABAergic signaling. The cartoon depicts the intracellular and extracellular compartments of a leukocyte, separated by the lipid bilayer of the cytoplasmatic membrane. **1** Extracellular/exogenous stimuli and intracellular/endogenous stimuli impact GABA signaling in leukocytes and exert paracrine and endocrine effects, respectively. **2** In GABAergic immune cells, GABA is enzymatically synthesized by glutamate decarboxylase (GAD65/67). **3** GABA transporters (GAT) transport GABA out from leukocytes. It remains undetermined if GAT transport also mediates influx of GABA in leukocytes, as in neurons. **4** Cation-chloride (Cl^−^) transporters (CCC) include expression of members from the KCCC and NKCC families. By maintaining Cl^−^ homeostasis and ionic gradient, they can function as regulators of GABA signaling. NKCCs mediate coupled movement of Cl^−^, sodium (Na^2+^) and potassium (K^+^) into the cytosol across the plasma membrane (illustrated), while KCCs mediate outward movement of Cl^−^ and K^+^ (not illustrated). **5** GABA-A receptors (GABA-A R) are activated by GABA and efflux or influx of Cl^−^ takes place, depending on the intracellular Cl^−^ concentration maintained by CCCs. **6** GABA-A R activation can result in depolarization of the membrane, leading to calcium (Ca^2+^) entry into the cell by opening of voltage-dependent Ca^2+^ channels (VDCC). If GABA-A R activation results in hyperpolarization, VDCCs are inactivated. Influx of the second messenger Ca^2+^ can impact multiple signaling pathways and cellular functions. **7** GABA-B receptors (GABA-B R) are metabotropic G-coupled receptors activated by GABA. **8** In a number of leukocytes, there is evidence of considerable transcriptional regulation and modulation of GABAergic genes and GABA-related genes, which will determine elevated or reduced protein expression. **9** Immune cell effector functions affected by GABAergic signaling

A comprehensive characterization of myeloid mononuclear phagocytes of human and mouse origin recently demonstrated a conserved expression of GABAergic molecular components [34]. Phagocytes consistently expressed the five principal components of GABAergic signaling (Fig. 1), namely (i) GABA metabolism, (ii) GABA transportation and secretion, (iii) GABA-A R activation, (iv) GABA signaling regulation by CCCs, and (v) effector Ca^2+^ channel signaling by VDCCs (Table 1). Furthermore, in both human and murine DCs, GABA evoked GABA-A R-mediated currents [35], with characteristics of neuronal synaptic and extra-synaptic GABA-activated currents [21].

**Table 1:** GABA signaling components expressed by primary immune cells and implications in immune functions

Immune cell	Subtype	Species	GABA metabolism	GABA transport	GABA-A R subunits	GABA-B R subunits	GABA-A R regulation	VDCC	Associated effect or function	References
Dendritic cell	MoDC	Human	GAD67 GABA-T	GAT1, GAT2, GAT3	*α*4, *ρ*1, *ρ*2, *ρ*3		NKCC1, KCC1, KCC3, KCC4	1.2, 1.3, 1.4, 2.1, 2.2, 2.3, 3.1, 3.2, 3.3	Migration	[34]
	MDC	Human	GAD67 GABA-T		*α*6, *β*2, *θ*, *ρ*1, *ρ*2			1.4, 3.1	Migration	[35]
	BMDC	Mouse	GAD65 GAD67 GABA-T	GAT1, GAT2, GAT4	*α*3, *α*4, *α*5, *β*2, *β*3, *γ*1, *γ*2, *δ*, *ρ*1, *ρ*2		NKCC1, NKCC2, KCC1, KCC2, KCC3, KCC4	1.1, 1.2, 1.3, 1.4, 2.1, 2.2, 2.3, 3.1, 3.2	Migration chemotaxis	[9, 34–36]
Macrophage	MoDM	Human			*α*1, *β*1, *ρ*2				Autophagy	[37]
	BMDM	Mouse	GAD65, GAD67	GAT4	*α*2, *α*3, *α*4, *α*5, *α*6, *β*2, *β*3,* γ1*, *γ*2, *δ*, *θ*				Autophagy	[37, 38]
	Peritoneal	Mouse	GAD65 GAD67 GABA-T	GAT2, GAT4	*α*1, *α*2, *β*1, *β*3, *δ*, *ε*				Cytokine secretion, inflammation	[9, 38, 39]
Monocyte		Human	GAD67 GABA-T		*α*4, *α*6, *β*2, *γ*1, *ρ*2			1.2, 1.3, 1.4	Migration chemotaxis	[34, 40, 41]
Microglia		Human	GABA-T		*α*1, *α*3, *β*1	B1, B2			Cytokine secretion	[42]
		Mouse	GAD65 GAD67 GABA-T	GAT2, GAT4	*α*1, *α*2, *α*3, *α*4, *α*5, *β*1, *β*2, *β*3, *γ*1, *γ*2, *γ*3, *δ*, *θ*, *ρ*1, *ρ*2		NKCC1, NKCC2, KCC1, KCC2, KCC3, KCC4	1.1, 1.2, 1.3, 1.4, 2.1, 2.2, 2.3, 3.1, 3.2, 3.3	Migration	[43]
		Rat				B1, B2			Cytokine secretion	[44]
T cell	CD4, CD8	Human	GAD67 GABA-T	GAT1, GAT2	*α*1, *α*3, *α*5, *β*1, *β*2, *β*3, *δ*, *π*, *ρ*2			1.2, 1.3, 1.4	Proliferation, cytokine secretion	[41, 45–50]
		Mouse	GABA-T	GAT1, GAT2	*α*2, *α*3, *α*5, *β*2, *β*3, *γ*1, *γ*2, *δ*		NKCC1	1.1, 1.2, 1.3, 1.4, 3.1, 3.2, 3.3	Cytokine secretion, migration, inflammation	[9, 45, 51–53]
		Rat			*α*1, *α*2, *α*3, *α*4, *α*6, *β*2, *β*3, *γ*1, *θ*, *π*, *ρ*1, *ρ*2, *ρ*3					[45]
NK cell		Human	GAD67 GABA-T	GAT2	*α*3, *α*5, *α*6, *β*2, *γ*1, *δ*, *θ*, *ρ*1, *ρ*2		NKCC1, KCC1, KCC3, KCC4		Cytotoxicity	[54]
		Mouse	GAD65, GAD67 GABA-T	GAT2	*α*2, *α*3, *β*2, *β*3, *γ*1, *γ*2, *δ*, *ε*, *π*, *ρ*1, *ρ*2		NKCC1, NKCC2, KCC1, KCC2, KCC3, KCC4		Cytotoxicity	[54]
B cell		Human			*α*1, *α*3, *β*2			1.2, 1.3, 1.4	Ca^2+^ flux	[41, 48, 55]
Neutrophil		Human	GAD65 GAD67			B2		1.2, 1.3, 1.4	Chemotaxis	[41, 56]

Blank cells indicate no publicly available data

*MoDC* monocyte-derived dendritic cell, *MDC* myeloid DC, *BMDC* bone marrow-derived DC, *MoDM* monocyte-derived macrophage, *BMDM* bone marrow-derived macrophage, *GABA-A R* GABA-A receptor, *VDCC* voltage-dependent calcium channel

In phagocytes, GAD67 was identified as the principal GABA synthesizing enzyme, while the relative expression of GAD65 was low in murine cells and undetectable in human cells [34]. Upon infection challenge with coccidian parasites, the extracellular GABA concentrations raised dramatically. Together with GAD67 expression, this is indicative of cytosolic GABA synthesis and vesicle-independent secretion by transportation through GATs, in line with secretory pathways described in neurons [57, 58]. Nonetheless, the precise secretory pathways of GABA in phagocytes remain uncharacterized.

The expression of GABA-A R subunit types was diverse in phagocytes, in line with the expression diversity in neurons [59, 60]. Yet, the repertoires of GABA-A R subunits expressed by different phagocyte types were, in theory, sufficient to constitute functional channels: at least one *α*, one *β*, and one-third type of subunit, or homopentamer-forming *ρ* subunits. While the precise subunit constituents of pentameric GABA-A Rs in phagocytes remain unknown, a functional hierarchy among GABA-A R subunits was identified and is discussed below [34]. Importantly, phagocytes expressed CCCs, which regulate GABA-A R function. Specifically, expression of NKCC1 was linked to GABA-A R function in DCs.

Finally, phagocytes expressed a highly conserved repertoire of VDCC sub-types. Stimulation of DCs with GABA elicited Ca^2+^ influx transients in the cytosol, which was inhibited by GABA-R antagonism. A prominent role for the VDCC subtype Ca_V_1.3 was demonstrated in human and murine cells [34, 36]. Thus, both human and murine phagocytes express a fully functional GABAergic machinery.

### Motogenic effects of GABAergic signaling in phagocytes

Activation of the GABAergic system of phagocytes by challenge with coccidian parasites mediates activation of motility in vitro and migratory responses in vivo in mice. This migratory activation is termed hypermigratory phenotype [61, 62]. Selective pharmacological antagonism of GABA-A R subunits indicates implication of *α*, *β* and *ρ* subunits in migratory responses. Additionally, in human or murine DCs, the finding that gene silencing of specific subunits (*α*4, *β*3 and *ρ*) inhibit GABA-A R-mediated hypermotility, but not gene silencing of *α*3 and *ρ*2, indicates a hierarchy among GABA-A R subunits mediating migratory activation or redundancy of function between subunits [34]. Similarly, pharmacological antagonism and gene silencing of NKCC1 or the VDCC subtype Ca_V_1.3 impacted the migration of DCs in vitro and in vivo in mice [34, 36]. This underlines the importance of the sequential GABAergic signaling cascade for the migratory activation of phagocytes.

It is tempting to draw parallels between the migratory effects of GABA on phagocytes and the motogenic role of GABA in embryonic interneuron migration in the developing fetus [18]. Furthermore, GABA-A R signaling has newly been associated with cancer cell metastasis, for example pancreatic cancer and breast cancer [63, 64]. Future research needs to determine if the motogenic molecular components identified in phagocytes [34, 36] are also implicated in immunomodulation and in cancer cell metastasis. Thus, receptor sub-types or other GABAergic components may be targeted to modulate cellular processes of clinical relevance [65].

### Motogenic GABAergic signaling and chemotaxis: synergistic effects?

Upon GABAergic activation, DCs maintain their chemotactic responses mediated by chemokine receptor 7 (CCR7) and in response to the chemokines CCL19/21 [35, 66]. Interestingly, upon GABAergic inhibition, DCs responded with directionality in the chemokine gradient but at significantly reduced velocities of DCs, thereby reducing the overall chemotactic response. This indicates that GABAergic activation DCs primarily acts on the mechanisms of cell motility rather than on regulation of directionality [67, 68]. Thus, GABA/GABA-A R-mediated hypermotility and CCR7-mediated chemotaxis acted simultaneously and enhanced the migratory properties of DCs [35, 62]. Similarly, chemokinetic GABAergic signaling cooperated with other chemotactic cues for embryonic neuronal migration [69]. In other cellular systems, GABAergic inhibition reduced the chemotaxis of monocytes and neutrophils [40, 56]. Moreover, GABA-B R signaling has been implicated in cancer cell metastasis [70] and in the motility of human sperm cells [71]. It remains unknown if GABA can also act as a chemoattractant for homing of phagocytes.

### Role of GABAergic signaling in immune activation of phagocytes

Immunomodulatory and down-modulatory effects by GABA were described early in peripheral blood mononuclear cells [48] and in experimental autoimmune encephalomyelitis, a model for multiple sclerosis [9]. Furthermore, GABA modulated cytokine release by peripheral blood mononuclear cells [50]*.* Specifically, GABA treatment has been reported to reduce IL-6/IL-12 production in macrophages [39] and impaired phagocytosis of macrophages and monocytic cells [40, 72]. However, the GABA transporter GAT2 was recently linked to pro-inflammatory IL-1*β* secretion in peritoneal macrophages [38]. A recent study showed that activation or blockade of GABA-A Rs influenced the phenotypic characteristics of alveolar macrophages towards classical (M1) or alternative (M2) activation, respectively [73]. Moreover, GABA signaling has been associated with antimicrobial responses, activation of autophagy, and phagosomal activation in macrophages challenged with intracellular bacteria [37]. Thus, upcoming evidences indicate that parasites, bacteria, and viruses modulate GABAergic signaling in immune cells for survival [35, 37, 74].

Jointly, GABA has been attributed both inhibitory effects on phagocyte activation and pro-inflammatory functions. This is likely a reflection of the versatility of GABAergic signaling and that its effects may be contextual and related to specific types/sub-types of phagocytes or their activation state. Yet, the understanding of how GABA impacts different immune functions is partly hampered by limited knowledge on the expression of GABA receptor subsets in different phagocytes and how these are implicated in cell type-specific effector functions. It remains also unknown if activation occurs in response to ambient GABA gradients in physiological compartments or if secreted GABA by an autocrine or paracrine loop is required for receptor activation. In these settings, the identification of novel extra-synaptic GABA-A/B R modulators may prove useful to test the impact of GABA signaling on immune cell functions and inflammation [75–77]. One interesting perspective is also the acidifying action of GABA-A Rs on intracellular pH [78]. Because intracellular pH gradients can influence the migration of cells [79], the activation of the inflammasome, and cytokine secretion [80], this merits further investigation.

## Microglia

Microglia are, in fact, part of the mononuclear phagocyte system [29] and are discussed separately here based on their specialized functions in the CNS. Microglial cells originate from primitive hematopoietic precursors outside the CNS and become the resident phagocytes of the brain [81]. Microglia participate in immune surveillance by rapidly responding to tissue injury and inflammation, similar to macrophages in peripheral tissues [82]. In neuroinflammatory processes, microglia also mediate regulative interactions with the endothelium of the neurovascular unit [83]. Additionally, microglia are important for neuroplasticity processes, for example in structural modifications after ischemic and traumatic insults [84, 85].

### Microglia express a GABAergic machinery

Earlier studies showed that microglia can respond to GABA and GABA-A R modulators with electrophysiological currents [86]. Moreover, expression of GABA-B Rs was reported in activated rat microglia [44] and expression of GABA-T and 3 GABA-A R subunits (*α*1, *α*3, and *β*1) in human microglia [42]. A recent comprehensive characterization in murine primary microglia revealed the expression of a complete GABAergic machinery (Table 1).

### Functions linked to GABAergic signaling in microglia

Importantly, microglia secreted GABA and exhibited migratory activation upon infection challenge [43]. Thus, infectious challenge with *Toxoplasma gondii* activated migration of microglia though GABAergic signaling, similar to DCs. This reinforces the idea of a conserved motogenic GABAergic signaling machinery in phagocytes [34]. It also highlights a hypothetical interplay between microglia and leukocytes, which infiltrate the brain parenchyma during infection and inflammation [87]. Furthermore, these findings raise questions related to the alteration of GABAergic synapse signaling in the rodent brain upon *T. gondii* infection [88]. Moreover, GABA can suppress IFN-γ production of microglia through inhibition of inflammatory pathways mediated by NF-kB and p38 mitogen-activated protein (MAP) kinases [42]. Because GABA-A R signaling impacts MAP kinase signaling via VDCCs in DCs [68] and migratory responses in microglia were linked to the MAPK regulator 14–3–3 [89], it is plausible that MAPK signaling is key to the modulatory effects of GABA on microglia. Finally, GABA signaling negatively regulated the dendritic morphology of mouse retinal microglia, indicating an impact on the cytoskeleton [90] and in line with the ascribed motogenic effects of GABA [34].

## T cells

T lymphocytes mediate important adaptive immune responses and provide long-lasting immunity (memory T cells). As effectors of adaptive immunity, different subsets of T cells have crucial functions in cytotoxic responses (CD8^+^ T cells), regulatory responses (CD4^+^ helper T cells), and cytokine responses against infection and cancer [91].

### GABAergic signaling components expressed by T cells

T cells harbor GAD67, GAT1, GAT2, GABA-T, and GABA-A receptor subunits suggesting the presence of a GABAergic signaling system similar to the neuronal system. Similar to human mononuclear phagocytes and NK cells, human T cells have conserved the expression of GAD67, but not GAD65 (Fig. 1). The GABA-catabolizing enzyme, GABA-T, and GABA transporters, GAT1 and GAT2, are expressed by both murine and human T cells (Table 1). Whether GABA is synthesized cytosolically and secreted by transporters or packaged into vesicles for secretion remains undetermined. The reported expression of GABA-A R subunits and GABA synthesis enzymes varies between species and depends on cell activation status or experimental mode, and what drives this variation remains undefined [92]. For instance, as different T-cell subsets express different GABA-A R subunits, they may display different sub-types of GABA-A Rs with diverse pharmacological properties and effects [93, 94]. The strength and polarity of GABA-A R-activated chloride currents depend on the intracellular chloride concentration set by CCCs [17]. In murine T cells, only NKCC1 has been detected to date, but in human PBMCs (T cells constitute ~ 45–70% of PBMCs), NKCC1, KCC1, KCC3, KCC4 were detected [52, 95, 96]. One single subunit of GABA-B Rs (B1) was detected in human PBMCs and it remains enigmatic if functional GABA-B R homodimers can be formed in T cells [95].

### Roles of GABAergic signaling in T cells

GABA has been shown to suppress the proliferation of T cells and to inhibit immune responses through functional GABA receptors [50, 97, 98]. GABA-induced single channel and whole cell currents recorded with patch-clamp electrophysiology were abolished by GABA-A R antagonists indicating presence of functional GABA-A Rs in CD4^+^ T cells [50, 99]. GABA inhibited Ca^2+^ influx and transcriptional activity of NF-κB in anti-CD3-stimulated human PBMCs and mouse splenic T cells in a GABA-A R-dependent manner [48, 98]. GABA and diazepam, a positive allosteric modulator of GABA-A Rs, inhibited IFN-γ production in anti-CD3 stimulated human and murine CD4^+^ and CD8^+^ T cells [100, 101]. When the gene coding for NKCC1, a GABAergic signaling regulator, was silenced, ablated, or pharmacologically antagonized, the migration and chemotaxis of murine T cells was inhibited [52].

Furthermore, GABA inhibited the proliferation T cells, and directly or indirectly impacted the secretion of up to 47 different cytokines from PBMCs derived from type 1 diabetes patients [50]. In mice, the onset of type 1 diabetes was delayed presumably by a reduction of T-cell responses, which improved the survival of pancreatic β cells [98, 101–103]. In a murine autoimmune encephalomyelitis (EAE) model, the GABA levels in serum and expression of GABA signaling components GAD, GAT1, GABA-T, and GABA-A receptor subunits in splenic T cells were downmodulated [51, 104]. Additionally, GAT1 knock-out mice exhibited aggravated EAE, enhanced splenocyte proliferation, and inflammatory cytokine production, suggesting dysregulation of GABAergic signaling in multiple sclerosis [51]. GABA also impacted T-cell responses in rheumatoid arthritis [105] and psoriasis [106].

#### VDCC components and functions in T cells

Only L-type VDCCs have been detected in human T cells to date, whereas in murine T cells, both L-type and T type VDCCs were described (Table 1). However, human PBMCs expressed transcripts for all sub-types of VDCCs [107]. The VDCC subtype Ca_V_1.4 contributed in T-cell receptor (TCR) activation, in the development and survival of naïve T cells and knocking out Ca_V_1.4 or blocking L-type channels inhibited TCR-induced Ca^2+^ influx, IL-2 production and proliferation of T cells [108–110]. Ca_V_1.1 channels were also shown to contribute in TCR-induced Ca^2+^ influx [111, 112]. Among the T helper cell sub-types, specifically murine Th2 cells, but not Th1 cells, expressed Ca_V_1.2 and Ca_V_1.3 channels. In a murine model of asthma, gene silencing of L-type VDCCs led to inhibition of TCR-induced signaling and cytokine secretion by Th2 cells, resulting in reduced inflammation and hyperactivity in lungs [41, 113]. In murine CD4^+^ T cells, Ca_V_3.1 channels were functionally active at resting membrane potential and drove Th17 cell cytokine responses but did not contribute in TCR-induced or store-operated Ca^2+^ entry (SOCE) [114].

Taken together, the data indicate that T cells harbor yet unidentified components of GABAergic and Ca^2+^ signaling machineries which regulate cellular functions such as proliferation, cytokine production, anti-inflammatory responses and Ca^2+^ homeostasis of T cells. In this context, the putative roles of store-operated Ca^2+^ (SOC) channels and other ion channels, for example potassium channels, need to be addressed due to their implication in various T-cell functions, including T-cell activation [115]. Because GABA-A R activation in T cells and other immune cells leads to changes in cell membrane potential, this may impact the function of SOC and potassium channels. Additionally, cross-regulation between VDCCs and SOCE may take place [116, 117]. Interestingly, GABA inhibits Ca^2+^ influx in T cells [98, 102], while GABA induces Ca^2+^ influx in phagocytes/DCs [34, 36]. These, seemingly contraposed effects of GABA, may hypothetically be explained by depolarization-mediated inhibition of SOCE in T cells and depolarization-mediated opening of VDCCs in phagocytes, as suggested in neurons [118]. In line with this assumption, artificial depolarization with KCl led to Ca^2+^ influx in DCs but not in T cells [36, 119, 120]. Alternatively, the relative expression of NKCCs and KCCs may differ in these two cell types, thereby regulating the depolarizing or hyperpolarizing action of GABA-A Rs, as shown in interneurons [18]. Jointly, the Ca^2+^-related immunomodulatory effects of GABA on T cells and other immune cells need to be further explored.

## NK cells

NK cells are effector lymphocytes of the innate immune system that mediate important responses against tumors and microbial infections [121]. NK cells have cytotoxic effects on target cells through perforin-dependent mechanisms or by inducing death receptor-mediated apoptosis. They also secrete cytokines that are pivotal for immunomodulation and are implicated in the regulation of T-cell-mediated responses. However, GABAergic signaling in NK cells has until recently remained unexplored [92]*.*

### Expression of GABAergic signaling components by NK cells

A recent report established that both human and mouse NK cells synthesize and secrete GABA, and express a GABAergic signaling machinery [54]. This includes GABA synthesis and degradation enzymes, GABA transporters, GABA-A R subunits, and CCCs, which can regulate GABA signaling (Table 1). Moreover, both human and mouse NK cells transcriptionally expressed repertoires of GABA-A R sufficient for the formation of heteropentameric (2*α*:s + 2*β*:s + 1 additional subunit) and homopentameric (*ρ*:s) GABA-A Rs. The *α*3, *β*2, and *ρ*2 subunits were most commonly expressed by tested human donors. NKCC1 was the principal CCC expressed and thus putatively implicated in the regulation of the direction of Cl^−^ flux mediated by GABA-A R activation. For GABA synthesis, murine NK cells expressed both GAD65 and GAD67, similar to murine microglia [43]. In contrast, human NK cells exclusively expressed GAD67 indicating a key role for this enzyme in GABA production. Related to transportation of GABA, only transcripts of GAT2 were detected in both human and murine NK cells. This contrasts with the expression of GAT2 and GAT4 by murine microglia and DCs [35, 43] and GAT1 was dysregulated in T cells [9, 51]. The expression of GAT2, jointly with GAD67 expression, indicates that GABA is synthesized cytosolically and secreted in vesicle-independent fashion for tonic modulations of GABA-A Rs in NK cells, as described in neurons [57, 58, 122]. Moreover, the reciprocal upregulation of GAD67 and downregulation of GABA-catabolizing GABA-T upon infection was consistent is human donors, indicating a tightly regulated GABA production in NK cells.

### Impact of GABAergic activation on NK cell effector functions

Importantly, in an infection challenge model, GABAergic activation in NK cells impacted their effector functions and interactions with DCs [54]. Upon challenge with *T. gondii*, NK cells responded with GABA secretion. Importantly, GABA secreted by parasitized NK cells (and DCs) hampered cytotoxicity and degranulation of NK cells in vitro. Additionally, GABA secreted by NK cells also modulated the migratory responses of DCs. GABA modulates cytokine release by peripheral blood mononuclear cells and T cells [50], and T-cell cytotoxicity [123], but its effects on NK-cell function have remained unclear [124]. Hypothetically, GABA may exert dual effects upon infection and inflammation: down-modulate pro-inflammatory responses and enhance DC migration [54]. In the context of infection in tissues, this dual effect may dampen inflammation but also modulate NK-DC interactions.

The precise mechanisms of downmodulation of NK cell responses by GABA remain uncharacterized. However, it was recently shown that GABA signaling is linked to MAP kinase activation in DCs [68] and MAP kinases regulate cytokine responses [125], which are inhibited by GABA in T cells [50]. Thus, it is likely that the immunomodulatory effects of GABA on NK cells are mediated by MAP kinase signaling. Additionally, future investigations need to address if GABAergic signaling acts on NK cells via effector VDCCs, as shown in DCs [34].

## B cells and granulocytes

To date, little is known about the expression of GABAergic components by B cells and granulocytes (neutrophils, eosinophils, basophils, and mast cells), which carry out crucial adaptive and innate immune functions, respectively.

Experimental evidence of functional GABAergic signaling by B cells is missing. However, in human B-cell lysates, western blot signal corresponding to GABA-A R *α*1 subunit was detected and GABA-A R *α*3, *β*2 subunit mRNA was amplified from human irradiated B cells [48].

In neutrophils, GABA-B Rs have been attributed a role in chemotaxis and been associated with neutrophil recruitment to inflammatory sites [56, 126, 127] (Table 1). Evidence of GABAergic expression and mechanistic studies are missing for eosinophils and basophils. However, *Gabra4* knock-out mice exhibited increased eosinophilic lung infiltration [128] and GABA antagonism decreased eosinophils in bronchoalveolar lavage in murine asthma models [129], indicating direct or indirect implication of GABA.

Thus, compelling evidence or functional data for GABAergic signaling in B cells and granulocytes are at present scarce or absent. However, the data indicate expression of GABAergic components or responsiveness to GABA, further underscoring the general expression of GABAergic system in cells of the immune system.

## Perspectives

The amino acid GABA is not incorporated into proteins. Instead, GABA serves as a signaling molecule and metabolic molecule in prokaryotes and eukaryotes. In the evolved vertebrate CNS, GABA has developed into an essential neurotransmitter. It is now clear that novel biological functions can be attributed to this versatile molecule. Given its expression and diverse functions in leukocytes, it is likely that GABAergic signaling is conserved throughout the immune system. The diversity of expressed GABAergic components in immune cells is likely also an indicator of yet undiscovered functions in the immune system. Recently emerged immunomodulatory functions of GABA include cytokine secretion, proliferation, cytotoxicity, migration and chemotaxis (Fig. 1, Table 1). The impact of GABA on phagocyte migration is in fact reminiscent of the motogenic role of GABA for embryonic interneuron migration in the developing fetus [18]. Furthermore, the putative impact of GABA on crucial interactions between immune cells needs to be explored, because it could open up for novel immunomodulatory approaches. These include, for example, the interactions between antigen presenting cells and T cells, between T and B cells in adaptive immune responses, or cytotoxic NK- and T-cell responses [130].

However, GABA is not only an intercellular signaling molecule between leukocytes but can also be considered an interspecies signaling molecule in host–microbe interactions. Recent reports show that bacteria, protozoan parasites and viruses modulate GABAergic signaling in immune cells for survival and colonization, including hijacking of leukocyte migration [34, 35, 37, 74]. These findings also raise the question whether microbial GABA or its metabolites are detected by sensing pathways of the immune system that detect specific dietary and microbial metabolites [131].

From a clinical perspective, GABA signaling has newly been associated with cancer metastasis [63, 64], for instance pancreatic cancer, breast cancer and gliomas [8, 10, 132]. Furthermore, the implication of GABA signaling in various autoimmune diseases, such as multiple sclerosis [9], type I diabetes [11, 50] and rheumatoid arthritis [105], indicates a general role in inflammatory responses. Future research needs to address if the motogenic effects in leukocytes are also implicated in inflammatory responses and in cancer cell metastasis. Hypothetically, receptor sub-types or other GABAergic components may be targeted pharmacologically to modulate migration and inflammatory responses of GABAergic cells [65].

The multiple points of interaction and communication exist between the CNS and the immune system have become increasingly evident [133]. Understanding neuro-immune interactions have not only advanced our understanding of immunity but also identified new therapeutic possibilities in inflammatory and autoimmune disease. From this perspective, the biology associated with GABA and other neuroactive molecules in immune cells represents an emerging field.

## Data Availability

The datasets used and analyzed in this study are available from the corresponding author on reasonable request.
